# User experience and acceptance of patients and healthy adults testing a personalized self-management app for depression: A non-randomized mixed-methods feasibility study

**DOI:** 10.1177/20552076221091353

**Published:** 2022-04-07

**Authors:** Gwendolyn Mayer, Svenja Hummel, Neele Oetjen, Nadine Gronewold, Stefan Bubolz, Kim Blankenhagel, Mathias Slawik, Rüdiger Zarnekow, Thomas Hilbel, Jobst-Hendrik Schultz

**Affiliations:** 1 Department of General Internal Medicine and Psychosomatics, 27178 Heidelberg University Hospital, Heidelberg, Germany; 2 Information and Communication Management, 26524 Technische Universität Berlin, Berlin, Germany; 3 medisite GmbH, Hanover, Germany; 4 38932 Westphalian University of Applied Sciences, Gelsenkirchen, Germany

**Keywords:** eHealth, apps, personalized medicine, self-monitoring, personalized medicine, depression, psychology, anxiety, psychology, telemedicine, mixed methods

## Abstract

**Objective:**

Previous studies have shown positive treatment outcomes of e-mental health applications targeting depression. However, few applications provide personalized features. The aim of the present study is to ask for the user experience and acceptance of patients with depression and healthy adults, who tested the self-management app Self-administered Psycho Therapy SystemS over a period of 5 days. The results serve as a source for evidence-based recommendations for developers and clinicians.

**Methods:**

A total of 110 participants (41 patients and 69 healthy controls) tested the app Self-administered Psycho Therapy SystemS over a period of 5 days and completed evaluation sheets developed for the purpose of this study. Quantitative measures were asked with 5-point Likert-scaled items (range: −2 to + 2) for the perceived quality of the programme and its components, its practicality (both referred to as user experience) and its acceptance. Student’s *t*-tests and Pearson correlations were calculated for group comparisons and associations, respectively. Open text fields were analysed by applying a qualitative structuring content analysis.

**Results:**

The perceived quality of the total programme was rated with M = 0.96 (SD = 0.82), the practicality was M = 0.84 (SD = 0.08) and the acceptance was M = 0.25 (SD = 1.04). Patients rated perceived quality of the total programme and acceptance higher than healthy adults, while there was no difference in practicality. Acceptance was associated with increased depression scores (r = 0.33, *p* = .01), higher scores of perceived quality of the total programme (r = 0.48, *p*< .001) and of practicality (r = 0.45, *p* < .001). Feedback of both groups regarding usability, therapeutic content and personalization revealed a strong wish for guidance and insights into mood progress, opportunities for choice of interventions and features of customization for individualized treatment.

**Conclusions:**

Patients with depression accepted the app Self-administered Psycho Therapy SystemS more than healthy adults and gave higher ratings in quality. User experience of all users shows a need for features of guidance, choice and personalization that clinicians and developers of future apps should pay attention to.

## Introduction

### Increasing relevance of e-mental health care

Many efforts have been made to support mental health with the help of web-based or computerized interventions, mainly referred to as the so-called e-mental health apps that range from short smartphone-based self-monitoring apps to extensive e-health programmes lasting several weeks. The pandemic of COVID-19 even accelerated this development, as e-mental health apps deliver fast and location-independent support to the general population suffering from contact restrictions on the one hand and patients with pre-existing mental disorders on the other hand.^1–4^

The growing relevance of e-mental health care is emphasized by two sociopolitical developments: First, the awareness and acceptance of experts towards digital mental health support have significantly increased in recent years,^5,6^ which resulted in the integration of telemedical support into legal frameworks, as realized in Germany in the recently launched law on digital supply in medicine,^7,8^ and in France.^9^ Additionally, professional associations like the Anxiety and Depression Association of America (ADAA) recommend quality assured apps for mental health support.^10^ Second, the range of e-mental health support is extending and the interventions get more and more specific, tackling syndromes such as perfectionism,^11^ skin picking^12^ or fibromyalgia.^13^

### Current status of e-mental health care for depression and anxiety

A scoping look at the first reviews of e-mental health apps reveals that the first attempts to deliver online support from 2005 to 2010 targeted depression and anxiety.^14^ Since then, the programmes have become more and more elaborated and their positive treatment effects have been shown several times.^15,16^

A major share of currently available e-mental health apps for depression (85%) makes use of digital cognitive behavioral therapy (CBT) approaches,^17^ but not all of them follow the treatment guidelines that are published in therapy manuals.^18^ Most of the programmes use CBT interventions that show a highly structured format, such as activity planning, and are suitable to be digitized. Other psychotherapeutic methods such as interpersonal or psychodynamic approaches are so far underrepresented except those regarding guided psychodynamic self-help interventions.^19–21^ Common interventions found in current depression apps relate to journal keeping/diary, mood tracking, relaxation, behavioural activation, psychoeducation and cognitive restructuring.^18,22^ Various apps and programmes consider both depression and anxiety. Typical elements in anxiety apps are related to CBT as well, such as mood tracking, structuring techniques and exposure therapy.^23^ A major share of available anxiety apps employs relaxation, breathing and meditation techniques as well as mindfulness-based interventions.^24^

E-mental health apps differ in their degree of therapist involvement that ranges from no support, that is, self-guided programmes, to regular contact via e-mail, chat or videoconferencing systems.^25^ Guided interventions were found to be superior to unguided interventions in effectively reducing symptom severity in anxiety disorders.^26^ A recent network analysis with CBT interventions for the treatment of depression using different therapy delivery formats showed that unguided self-help formats were less effective than face-to-face or guided formats but superior to waitlist control conditions.^27^ Similarly, the usage of unguided interventions results in a significant reduction of depressive symptoms when compared to waitlist, no-therapy and placebo.^28^ However, the sole use of unguided interventions for depression and anxiety may suffer from low attrition rates.^29^ Personalization of features can contribute to an increase in adherence.

### Personalization in e-mental health apps for depression and anxiety

Evidence has been provided that depressive symptoms alter the perception of websites' usability, aesthetics and content.^30^ As cognitive functions may be impaired during a depressive episode, the receptivity to web-based information can be reduced, which highlights the necessity of an easy and understandable information delivery.^31^ The benefits of personalization for treatment adherence have been shown even in face-to-face interventions for adults.^32^ However, e-mental health apps show a great potential of personalized features, such as text messages and individualized feedback provided by the software.^33^

Personalization in e-mental health apps means that the generated content is targeted at the needs of the individual user based on their responses.^34^ Moreover, personalization makes use of contextual data collected by smartphones, which refers to an ecological momentary assessment (EMA) and has been shown to have therapeutic potential, especially in depressed individuals.^35^ By this, individualized content can be suggested via a text message at the most appropriate time according to behavioural patterns of the respective user. In sum, personalization may serve as a strategy to provide a tailored health communication to increase the effectiveness of a treatment.^36^

However, currently available depression apps rarely suggest personalized content on the basis of mood tracking, they rather provide generic templates in the delivery of interventions.^22^ A review regarding recent CBT apps for depression by Stawarz et al.^37^ highlights that therapists, as well as patients, appreciate the possibility of customization, nevertheless only 2 of the 31 tested apps offered these features. Finally, text messages that are sent to the users rather intend to motivate by promoting positive emotions than to suggest individualized interventions.^38^

### Instruments of mood tracking in e-mental health apps

Basic requirements for personalized features in e-mental health apps are a regular mood assessment and symptom monitoring. Applying these, a regular, situation-specific longitudinal information on patient’s progress can be provided.^39^ If e-mental health apps make use of mood tracking, they recur on validated psychometric scales, such as Patient Health Questionnaire-9 (7PHQ-9), Beck Depression Inventory-II (BDI-II), Geriatric Depression Scale or M3 Questionnaire.^40^ However, the repeated, daily use of the same questionnaire may not be useful, as patients get bored over the time and won't answer precisely any longer.^41^ Besides, the PHQ-9 asks for symptoms observed in the last 2 weeks and is recommended for follow-up measurement during treatment but not for daily use.^42^

In spite of a growing market and increasing offers, the technological sophistication of the systems remains low in the last decade, as a recent study has shown by reviewing e-mental health apps for major depressive disorder released in 2000–2017.^17^ The authors state that the majority of the applications rely on systems that are limited to merely presenting information, instead of interpreting data and acting upon generated knowledge, for example, by delivering content appropriate to the specific indication. As a result, the information gathered by symptom monitoring is reported back to the user without automatically interpreting data and delivering content referring to the data.

A summarizing look at the literature shows the availability of several evidence-based e-mental health apps targeting depression, both with and without comorbid anxiety. But until now, no app offers personalized help based on a detailed daily mood tracking with different non-repeating questions that are developed for a longitudinal use.

### Objective of the study

The present study aims to assess the user experience and acceptance of adult patients and healthy adults regarding the self-monitoring app Self-administered Psycho Therapy SystemS (SELFPASS), its self-assessment and its individual interventions. The main target is to provide evidence-based recommendations for developers and clinicians regarding the graphical and conceptional design of a self-management app for patients with depression, both with and without anxiety symptoms.

## Methods

### Study design

We conducted a cross-sectional study with a 5-day observational period, in which adult patients and healthy adults tested the app SELFPASS on a tablet or desktop computer. We used daily evaluation sheets that were developed for the purpose of the study.

### The app SELFPASS

The mobile app SELFPASS was developed in the similarly named research project, funded by the German Federal Ministry of Research and Technology. This application was designed to improve the self-management of patients with depression on the basis of an individualized daily mood score. The target group is patients with a diagnosis of depression who usually are waiting for a long period of time for a face-to-face psychotherapy.^43^ The app delivers a daily monitoring of depressive symptoms on the one hand and daily interventions to support the patients on the other hand. The app SELFPASS does not claim to replace a real psychotherapy but to support patients during waiting time, in order to bridge the treatment gap.^44^

A SELFPASS-specific item pool of mood-related questions was developed to cover all aspects of depressive symptoms according to the national disease management guidelines.^42^ The item pool has been validated and is described elsewhere.^45^

The programme starts with a basic self-assessment on the first day that consists of 42 questions. From the next day on, a daily self-assessment has to be completed with six questions at minimum, before the user can interact with the other parts of the application. The number of daily questions depends on the answers of previous days, as the algorithm pursues single symptoms when a critical score is exceeded and delivers contact information in case of acute suicidality. Based on the self-assessment, an individualized symptom score is calculated that the programme uses to recommend three suitable interventions, of which one should be chosen by the user per day. The rationale of the suggestions for interventions is based on the diagnostic procedure of International Classification of Diseases 10th Revision (ICD-10) symptoms for a depressive disorder. Following ICD-10 core symptoms are depressed mood, loss of interest, and loss of energy. Additional symptoms refer to lack of concentration, feelings of worthlessness, guilt, pessimistic future expectations, suicidal ideation, sleep disturbances and loss of appetite.^46^ All interventions were previously assigned to single symptoms. Details are shown in the online Supplemental material.

The app reminds the user of completing the daily self-assessment after starting the programme. However, the app can be started at any time of the day.

**Figure 1.**
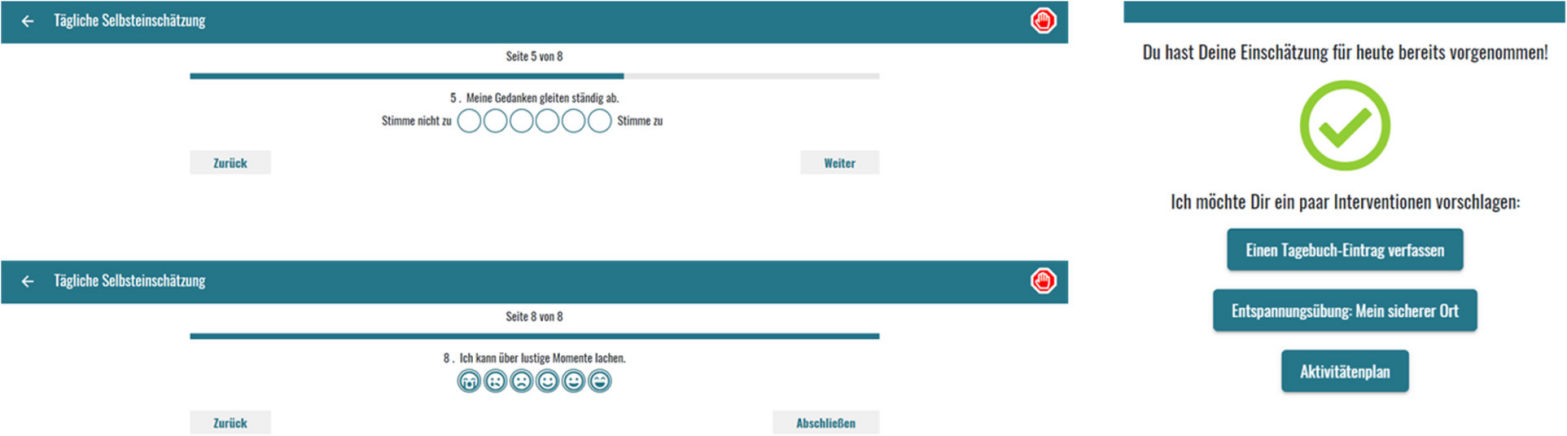
Examples for the daily self-assessment (above: ‘My thoughts always drift away’; below: ‘I can laugh about funny things’). Right: Personalized suggestion of interventions (‘You have finished your self-assessment for today. I want to suggest the following interventions: Make an entry into your diary, Relaxation exercise: My safe place, Activity planning’).

Examples of the graphical design of the daily self-assessment and the intervention recommendations are shown in Figure 1.

### SELFPASS interventions

A set of common interventions of CBT and integrative approaches were digitized. The interventions do not follow a specific order, as the main characteristic of the app is making individualized propositions on the basis of the daily symptom score. Four interventions were adopted from guidelines and manuals of CBT^47–49^: journal keeping, behavioural activation, thought stop and cognitive restructuring (‘switch off your radio of negative thoughts’). Behavioural activation is a commonly used intervention for depressed patients that seek to help them get back into a daily structure of activities. For this, a calendar is provided that allows for adding pleasurable, neutral and unpleasant activities that are planned for the day. The intervention ‘thought stop’ includes a short audio file that helps stopping unhelpful or harmful negative thoughts. The user is then invited to press a ‘stop’ button designed similar to the traffic sign. Cognitive restructuring presents typical crucial everyday situations with three options to react with more or less helpful thoughts out of the cognitive distortions according to Beck.^47^

**Figure 2.**
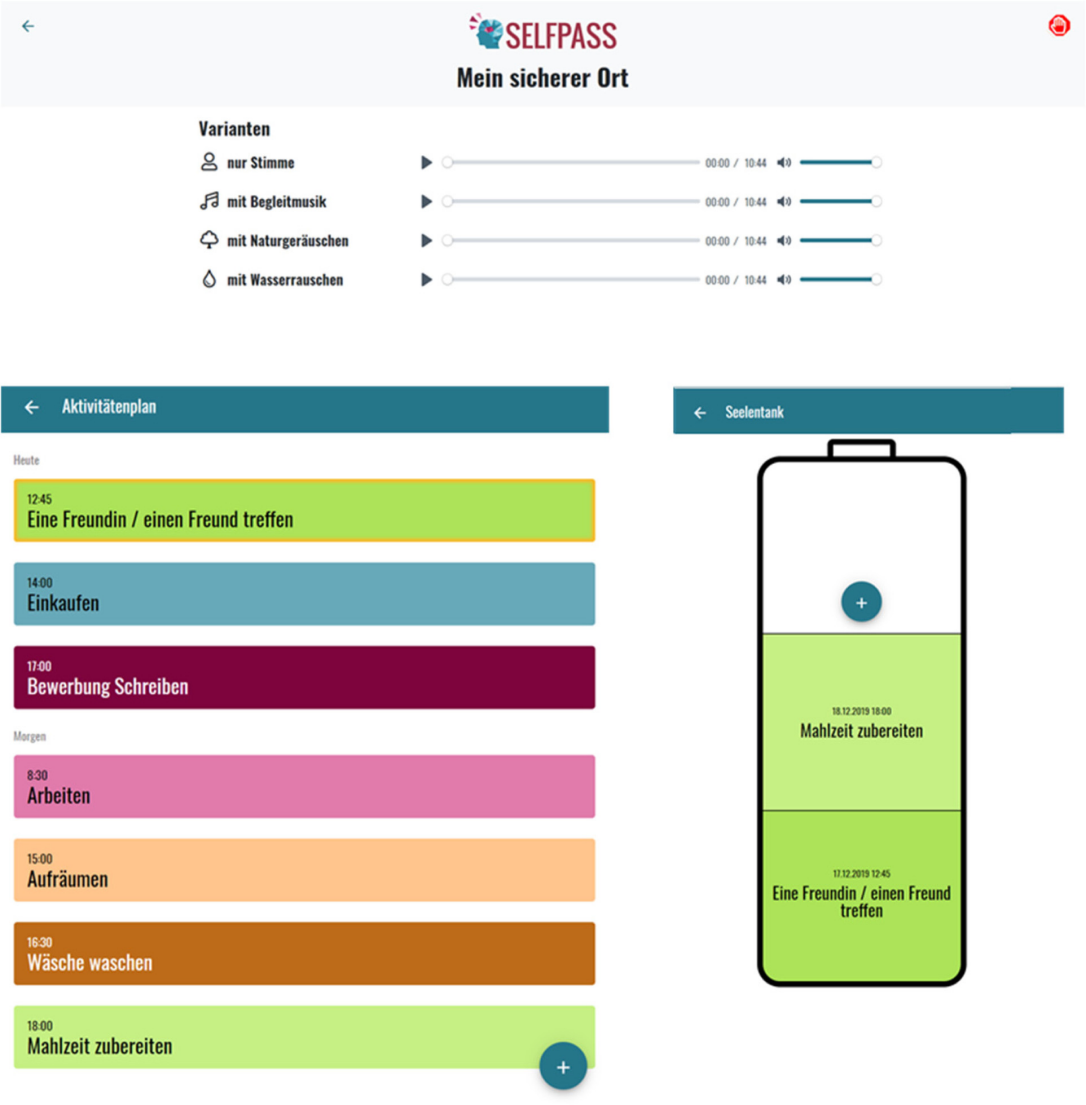
Examples for interventions. Top: Relaxation exercise ‘My safe place’ (‘voice only’, ‘with background music’, ‘with sounds of nature’, ‘with sounds of water’. Left: Activity planning (top down: ‘meet a friend’, ‘go shopping’, ‘write an application’, ‘work’, ‘clean up’, ‘do the laundry’, ‘prepare a meal’). Right: Soul tank (top down: ‘prepare a meal’ and ‘meet a friend’).

Moreover, interventions from general psychotherapeutic approaches include a concentration exercise and an album of strengths and weaknesses^49^ that allows for compiling an individualized profile of personal positive characteristics and shortcomings, while reflecting at the same time the respective weakness that might be part of the strength and vice versa. Three modules of psychoeducation provided information regarding depression, sleep behaviour and anxiety. Another intervention was called ‘soul tank’.^49^ The soul tank, which is designed as a battery, can be filled either directly by planning a positive activity in the intervention or indirectly by doing this in the behavioural activation intervention (Figure 2). Four relaxation exercises are included based on the respective symptom score (‘My safe place’, ‘Laying down a burden’, ‘Meeting my inner power’, ‘Making peace with myself’). These exercises are provided as an audio file with a short introduction at the beginning. Finally, two interventions target anxiety: A temporary calming exercise (‘Come to the ground’) and a coping intervention derived from the therapeutic method of writing letters to oneself.^50^

A previous study already investigated the theoretical constructs of usability, trust, task technology fit, attitude and intention-to-use of SELFPASS with patients and students;^51^ however, a deeper understanding of the user experience, perceived practicality, and acceptability at the level of single interventions is still missing. These insights will deliver recommendations for app developers and clinicians working on similar web-based interventions for depression and anxiety regarding usability, therapeutic content and personalization, which is considered to be a key criterion for the adherence to the programme.

### Recruitment and procedures

The study participants were recruited in two stages from February to May 2019 and from June to August 2020. We included patients and healthy controls, as results from the literature reveal important differences in the perception of web-based information delivery due to differences in cognitive functions.^31^ The patients were recruited in the Heidelberg University Hospital. All patients suffered from mild to moderate depressive symptoms during the past year. The patients were recruited in inpatient and outpatient clinical settings of the Department of Internal Medicine and Psychosomatics that provides a targeted concept of individual and group-based psychotherapy; therefore all patients had significant experiences in psychotherapeutic methods. Healthy adults that took part in the study were students at the Berlin Institute of Technology and volunteers recruited via contact networks of the Heidelberg University Hospital.

All participants got an individual account to the SELFPASS web application and were asked to test the programme for a time period of five subsequent days. The daily expenditure of time was between 20 and 30 min.

Ethical approval was obtained by the Ethics Commission of the Medical Faculty of Heidelberg (S-296/2018) prior to data collection. The study ‘Feasibility study: Checking the functionality, practicality (P) and acceptance (A) of the app SELFPASS’ was registered by the DRKS (Deutsches Register Klinischer Studien, German Clinical Trials Registry; DRSK-ID: DRKS00015614).

Besides demographic data, we asked the participants to rate their computer skills on a 5-point Likert scale from ‘not sufficient’ to ‘very good’. Additionally, we asked all participants to complete a mood assessment using the depression module of the PHQ-9. When interpreting the cut-off values of the PHQ-9, according to the manual a mean score of 0–4 is rated as ‘none-minimal’, 5–9 ‘mild’, 10–14 ‘moderate’, 15–19 ‘moderately severe’ and 20–27 ‘severe’ depression.^52^

### Evaluation criteria

A mixed-methods approach was applied to assess the user experience and the acceptance of SELFPASS and its interventions. According to the ISO standard, DIS 9241-210:2008 user experience refers to ‘a person's perceptions and responses that result from the use or anticipated use of a product, system or service’.^53^ Out of various ‘acceptance’ definitions, we chose a pragmatic one, that focuses on the willingness to use a system based on either theoretical knowledge or experiences.^54^ Therefore, we regard perceived quality (PQ) of the system and its components and practicality as necessary prerequisites to assess the user experience that may result in acceptance (A). We further asked for user experience in open text fields. The strategy of analysis is reported separately (see ‘qualitative analysis’).

After completing the daily self-assessment (the basic self-assessment on the first day, respectively), several questions referred to the PQ of this feature. After that, the participants tested one intervention per day and the questions referred again to quality criteria of the intervention tested, respectively. On the last day, all participants were asked to make an assessment regarding the perceived quality of the total programme (PQTP). We defined P as suitability for daily use and evaluated this characteristic after each particular intervention and, on the last day, regarding the whole programme. The acceptance was also measured on the last day. All items were presented as a 5-point Likert scale from ‘does not apply at all’ to ‘applies completely’. Items with a negative polarization were recoded. The quantitative items with the related scores are listed in Table 1.

**Table 1.** Quantitative measures referring to the outcome variables user experience and acceptance.

Outcome	Score	Item
User Experience	PQSA: Perceived quality of the basic self-assessment/daily self-assessment (BSA/DSA)	The questions of the BSA/DSA were easy to understand.
		Doing the BSA/DSA was easy.
		The results of the questions were clear and transparent.
		I feel that I am taken seriously by the questions of the BSA/DSA.
	PQI: Perceived quality of the respective intervention	The intervention was too short.^a^
		The intervention was too long.^a^
		The intervention was easy to understand.
		The background information of the intervention was provided sufficiently.
		I was bored by the intervention.^a^
		The intervention is helpful for coping with my illness.
		I feel that I am taken seriously by the intervention.
	PQTP: Perceived quality of the total programme (day 5)	The structure of the programme was easy to follow.
		The graphical design of the programme addresses the subject appropriately.
		The background information about depression was provided sufficiently.
	P: Practicality	The intervention is suitable for daily use.
		I felt being bothered by the programme in my daily life.^a^ (last day only)
Acceptance	A: Acceptance	I have concerns about using a programme such as SELFPASS.^a^
		I can imagine using a programme such as SELFPASS.
		I can imagine buying a programme such as SELFPASS.
		If possible, I would ask my insurance to cover the costs for SELFPASS.

^a^ Reverse coding.

SELFPASS: Self-administered Psycho Therapy System.

### Statistical analysis

Data analysis was carried out using SPSS Statistics (version 24; IBM Corp, Armonk, New York, USA).^55^ The data of the two groups, adult patients and healthy adults, were recorded simultaneously. The range of all continuous items was coded from −2 to + 2. Descriptive statistics were performed by calculating frequencies, means, and standard deviations. We analysed group differences when appropriate by performing Student’s *t*-tests for mean differences of independent samples after testing for homogeneity of variances (VH). In case of categories, we calculated chi-square tests. We calculated Pearson correlations to assess possible relationships between the age, computer skills, and depression scores and the PQTP, the P and the A of the participants, independent from being a patient or a healthy adult. In all cases, we regarded a significance level of *p* = .05 and report the 95% CI when appropriate.

### Qualitative analysis

We found 1031 comments made by patients and healthy adults in open text fields and then analysed by means of structuring content analysis following Mayring.^56^ This method includes a deductive approach so that categories are built in advance to find a clear assignment of single text snippets to the categories. In the present study, we focused on the user experience and classified three main feedback categories: (i) the usability, (ii) the therapeutic content and (iii) the personalization of this content. We regarded ‘usability’ as an interactive feature that is based on the users’ perceptions to a certain extent, that is, as the ‘capability of the software product to be understood, learned and liked by the user, when used under specified conditions’ as defined in ISO/IEC 9126-1.^57^ For example, features of design, simplicity, ease of structure and guidance of the whole programme were regarded as usability. Statements regarding the assessment of an idea behind an intervention or their possible effects were categorized as therapeutic content. Personalization was coded in cases of suggestions to respect the users’ individual needs and circumstances. Some text snippets included more than one aspect; in this case, we applied multiple codings.

The qualitative analysis was done by two authors (GM and SH) and checked for agreement. We then collected positive feedback and suggestions to list already existing, expandable features. Negative, critical feedback towards the usability and the content was regarded as valuable information to guide further improvement of the features implemented in the application. The results will be reported exemplarily with the most salient statements for the total programme, the basic and daily self-assessment and each of the most frequently used interventions. They were compiled in a list of recommendations for future clinicians and developers that we provide in the discussion section of this paper.

## Results

### Participant characteristics

A total of 117 participants took part in the study, 7 of them were excluded because they did not complete any intervention. The final sample consisted of N = 110 participants, of which 41 (37.3%) were patients and 69 (62.7%) healthy adults. The age of all participants was M = 30.47 (SD = 11.46), the patients were M = 30.90 years old (SD = 12.97), the healthy adults M = 30.18 (SD = 10.43). Fifty-eight (52.7%) participants were female, 46 (41.8%) male. Further demographic characteristics are shown in Table 2.

**Table 2.** Demographic values of the sample (N = 110) and group comparisons.

Characteristics		Patients	Healthy adults	All participants	Significance
		n (%)	n (%)	n (%)	*P* value
**Age** (years), Mean (SD)		30.90 (13.0)	30.18 (10.4)	30.47 (11.5)	.76
**Gender**					.00^**^
	Male	11 (26.8)	35 (50.7)	46 (41.8)	
	Female	30 (73.2)	28 (40.6)	58 (52.7)	
	Missing	0 (0.0)	6 (8.7)	6 (5.5)	
**Family status**					
	Single	29 (70.7)	46 (66.7)	75 (68.2)	.66
	Married	5 (12.2)	13 (18.8)	18 (16.4)	.36
	Separated/Divorced	5 (12.2)	3 (4.3)	8 (7.3)	.13
	Widowed	1 (2.4)	0 (0.0)	1 (0.9)	.19
	Others	0 (0.0)	1 (1.4)	1 (0.9)	.70
	Missing	1 (2.4)	6 (8.7)	7 (6.4)	
**Employment**					
	Yes	18 (43.9)	28 (40.6)	46 (41.8)	.73
	No	23 (56.1)	35 (50.7)	58 (52.7)	.77
	Missing	0 (0.0)	6 (8.7)	6 (5.5)	.19
**Highest level of education**					
	High school	25 (61.0)	16 (23.2)	41 (37.3)	.00^***^
	College degree	12 (29.3)	45 (65.2)	57 (51.8)	.00^***^
	Dissertation/PhD	2 (4.9)	2 (2.9)	4 (3.6)	.59
	No degree	2 (4.9)	0 (0.0)	2 (1.8)	.06
	Missing	0 (0.0)	6 (8.7)	6 (5.5)	
**Computer skills**^a^**,** mean (SD)		1.02 (0.76)	1.42 (0.75)	1.27 (0.77)	.01^*^^b^
**Depression Score (PHQ-9),** Mean (SD)		12.45 (4.31)	4.15 (4.46)	7.68 (6.01)	.00^***^^b^
**Total**		41 (37.3)	69 (62.7)	110 (100)	

^a^ Range: −2 = insufficient, + 2 = very good.

^b^ In case of continuous variables: assumption of variance homogeneity are violated.

^*^ *p <*.05 (two-tailed).

^**^ *p <*.01 (two-tailed).

^***^ *p <*.001 (two-tailed).

### User experience and acceptance

#### Basic/daily self-assessment

The perceived quality of the basic/daily self-assessment (PQSA) was M = 1.40 (SD = 0.55). Patients rated the PQSA with M = 1.52 (SD = 0.37), healthy adults with M = 1.32 (SD = 0.62).

#### Interventions

The most frequently chosen intervention was the concentration exercise, which was done 129 times (patients: 50, healthy adults: 79), followed by the relaxation exercise, which was tested 102 times (patients: 46, healthy adults: 56). The diary was tested 70 times (patients: 32, healthy adults: 38), the behavioural activation intervention 43 times (patients: 20, healthy adults: 23), and the album of strengths and weaknesses 19 times (patients: 11, healthy adults: 8). Soul tank was chosen 14 times (patients: 11, healthy adults: 3), as well ‘Switch off’ (cognitive restructuring; patients: 6, healthy adults: 8), and all other interventions less than 2 times.

The PQ of the diary and the album of strengths and weaknesses was rated highest with M = 1.32 (SD = 0.51, N = 57) for the diary and M = 1.13 (0.62, N = 18) for the album. Further details and levels of statistical significance of group differences are presented in Table 3.

**Table 3.** Results of the outcome scores referring to the perceived quality (PQ), practicality (P) (both user experience) and acceptance (A) of SELFPASS, its self-assessment, intervention components and total concept together with results of Student’s *t*-tests.

Score	Level of Assessment	Patients	Healthy Adults	All Participants	Significance
	(N)	Mean (SD)	Mean (SD)	Mean (SD)	*P* value
PQSA	Basic/Daily Self-Assessment (110)	1.52 (0.37)	1.32 (0.62)	1.40 (0.55)	.04^a^
PQI	All Interventions (110)	1.02 (0.42)	0.81 (0.65)	0.89 (0.58)	.04^a^
	Concentration (79)	0.98 (0.54)	0.86 (0.62)	0.92 (0.58)	.36
	Relaxation (70)	0.97 (0.62)	0.77 (0.79)	0.87 (0.72)	.24
	Diary (57)	1.38 (0.42)	1.26 (0.59)	1.32 (0.51)	.38
	Behavioural Activation (37)	0.78 (0.87)	1.02 (0.72)	0.92 (0.79)	.37
	Album of Strengths and Weaknesses (18)	1.25 (0.57)	0.94 (0.69)	1.13 (0.62)	.31
	Soul Tank (14)	0.81 (1.09)	0.67 (0.64)	0.78 (0.99)	.84
	Switch Off (14)	0.77 (0.98)	0.61 (0.75)	0.68 (0.82)	.73
PQTP	Total (70)	1.17 (0.66)	0.76 (0.91)	0.96 (0.82)	.03^a^
Practicality	Programme	0.92 (0.67)	0.79 (0.90)	0.84 (0.82)	.37^a^
Acceptance	Programme	0.51 (0.86)	−0.01 (1.14)	0.25 (1.04)	.03

^a^ Assumption of variance homogeneity violated.

PQSA: perceived quality of the basic/daily self-assessment; PQTP: perceived quality of the total programme; SELFPASS: Self-administered Psycho Therapy SystemS.

#### Total programme

The PQTP was rated with M = 0.96 (SD = 0.82). Patients rated this aspect higher (M = 1.17, SD = 0.66) than healthy adults (M = 0.76, SD = 0.91). This difference was statistically significant (*P* = .03).

Patients rated the P of the programme with M = 0.92 (SD = 0.10) higher than healthy adults, who showed an M = 0.79 (SD = 0.90). The P of all participants was rated with M = 0.84 (SD = 0.08).

The A of the programme was rated higher by patients (M = 0.51, SD = 0.86) than by healthy adults (M = -0.01, SD = 1.14). The total A was M = 0.25, SD = 1.04. Again, this difference was statistically significant (*P* = .03).

### Relationship of age, computer skills, depressions scores and PQTP, P and A

The correlation of Patient Health Questionnaire (PHQ) depression scores with the acceptance of the programme was significant with *r* = 0.33, *P* = .01. As well the PQ of the programme and the perception of P correlated significantly with the A (*r* = 0.48, *P* < .001; *r* = 0.45, *P* < .001). Further details are shown in Table 4.

**Table 4.** Correlations of age, computer skills, depression score, perceived quality of the total programme (PQTP), practicality (P) and acceptance (A).

		Age	Computer Skills	Depression	PQTP	P	A
Age	*r*	1					
	*P*						
	*N*	102					
Computer skills	*r*	−0.28^**^	1				
	*P*	.00					
	*N*	102	107				
Depression	*r*	−0.01	−0.32^**^	1			
	*P*	.94	.00				
	*N*	90	94	94			
PQTP	*r*	−0.12	0.10	0.23	1		
	*P*	.35	.44	.08			
	*N*	65	68	57	70		
P	*r*	−0.11	0.02	−0.02	0.51^***^	1	
	*P*	.25	.83	.86	.00		
	*N*	102	107	94	70	110	
A	*r*	−0.04	−0.09	0.33^*^	0.48^***^	0.45^***^	1
	*P*	.76	.46	.01	.00	.00	
	*N*	65	68	57	70	70	70

^*^ Significant correlation with *p <*.05 (two-tailed).

^**^ Significant correlation with *p <*.01 (two-tailed).

^***^ Significant correlation with *p <*.001 (two-tailed).

### Qualitative results

The participants made many suggestions regarding the *therapeutic content*, the *usability* and needs for further *personalization* of the programme SELFPASS, the basic and daily self-assessment and the single interventions.

#### Feedback towards the total programme

Patients and healthy adults made 185 comments regarding the total programme (patients: 122, healthy adults: 63). Both groups appreciated the principal concept of the programme and its *therapeutic content*:

“At first glance, the app seems confusing, but then you notice that it has a lot of great and valuable content.” (Patient, 21, male)

“Find the app very extensive and informative. Helpful in everyday life, but a little more ‘red thread’ would improve it. Took some time to find my way around.” (Healthy adult, 44, male)

Some participants draw comparisons to other programmes and their former experiences:

“I’ve run into a wall with similar programs … it feels empty and strange soon, but it helps for a while. The question is always how long.” (Patient, 23, female)

Critical comments regarding the content referred to the need for a deeper understanding, how specific interventions help fighting depressive symptoms, over which time span they should be done and why they are recommended by the programme. The latter aspect refers as well to personalization features. Further, some participants pointed out that the fear of missing data security would prevent them from using the app.

However, the *usability* was assessed critically and many participants expressed the wish for more guidance on the one hand and gamification elements on the other hand. While some participants rated the design of the programme as ‘appropriate for the seriousness of the subject’, the majority of the users noted that the programme lacks in guidance and seemed confusing to them. They specifically missed advice to integrate the interventions into their daily life. Some comments showed that the users did not understand exactly when an intervention was finished and what should be done next.

In terms of *personalization*, the participants requested a broader variety of interventions that target specific mood states, for example, motivational interventions against hopelessness. One patient suggested:

“It would be useful to have a kind of database in which you can create statistics about your own mood. Perhaps it would be useful to interview again after the intervention to see whether the intervention is helpful in the short term.” (patient, 24, female)

#### Feedback towards the basic and daily self-assessment

In total, 255 comments on this topic were made by the participants, 134 of them were expressed by patients and 121 by healthy adults. The *therapeutic content* of the questions was assessed in a contradictive way: A considerable part of the participants rated the questions as clear, easy to understand, and following a logical order. Another part called for more variation in the questions. Some questions were felt to be too extreme and exaggerated; questions with a negative wording were felt to be too complicated to think about (e.g. ‘I am not easily distracted’). Some questions were assessed as too general, for example, regarding alcohol consumption or body weight.

*Usability-*related feedback expressed the wish for more automatic functionalities, for example, to be asked every morning for their mood and a presentation of the results. Participants noted to prefer Likert-scaled items rather than smileys, because the answer to the questions seemed to be clearer to answer with a scale from ‘do not agree at all’ and ‘totally agree’. Smileys were felt to be ‘childlike’ (patient, 36, female).

The comments that referred to *personalization* voiced the wish for the delivery of individual results: ‘With the daily self-assessment, I might have wished for a more tangible result. -> Where am I now? -> How strong is the depression in relation to personal events?’ (patient, 23, female). Transparency of data processing of the daily self-assessment was a key need for the feeling of being taken seriously.

#### Feedback towards the interventions

##### Concentration exercise

In total, 191 comments referred to the concentration exercise *sort letters*, 120 of them were made by patients, 71 by healthy adults*.* Especially patients were skeptical about its *therapeutic content*. Some comments showed a high level of awareness of own symptoms: ‘well, it supports my compulsion to do everything right’ (patient, 55, female). Moreover, some comments hint at a potential worsening of symptoms: ‘I don't know if such a concentration exercise would depress me in the long run, especially if the words don't come to my mind. This does not have a particularly positive effect on the state of mind’ (patient, 30, male), ‘A little consolation? I did really badly’ (patient, 55, female) and even one user in the group of healthy adults wrote to be ‘sad’, because ‘the words are sometimes complicated, I couldn't solve them’ (healthy adult, unknown age and gender). Users voiced the wish for transparency: ‘I would like to know how this exercise should help me focus on an ongoing conversation - but that might be too specific. But I would have liked a word or two on the subject of transfer’ (patient, 26, female). Comments that were coded as *usability* criticized that the intervention only accepted one possible solution as correct. Few comments referred to potential *personalization* features, for example, more options to choose or a more automatic solution that finds the right degree of difficulty needed by the respective user: ‘Recommendation: choose another concept, e.g., a programme that adapts the level of difficulty fluently depending on the errors or correct answers’ (healthy adult, 41, male).

##### Relaxation exercise

The 4 relaxation exercises were commented 178 times, 97 times by patients and 81 by healthy adults. Their *therapeutic content* was appreciated by many participants: ‘It was a good feeling to get involved in a dream place’ (healthy adult, 19, female), and ‘I came to rest well, without the program I would not have consciously taken the time for it’ (patient, 26, female). Critical voices expressed their doubts regarding the helpfulness of the intervention: ‘Personally, I cannot ‘save’ such a feeling of safety and security for everyday use. Nevertheless, it helps me at this moment and in general for everyday life’ (healthy adult, 22, male). Another patient wrote: ‘It helps to ground yourself briefly but doesn't make my negative beliefs go away’ (patient, 20, female). However, the content of the text was seen critically as well: Some users found the wording childlike, as one of the interventions used inner pictures like a flying carpet or a rocket. Another feedback pointed out: ‘In my illness, I cannot reach this inner strength so easily - at least not on days when I feel bad (as today)’ (healthy adult, 22, male). User statements that referred to the *usability* of this intervention claimed to have a better quality of the sound. Additionally, usability feedback referred to the length of the interventions. The four interventions were between 7 and 10 min each, which was rated in various ways from ‘too long’ to ‘adequate’ and ‘perfect’. Several ways of possible *personalization* features were described, for example, a female voice to choose, more different relaxation exercises to choose, more ways to choose the place and body position to relax, and a way of adapting the speed of the intervention.

##### Diary intervention

The diary intervention was assessed qualitatively 45 times by patients and 34 times by healthy adults (79 times in total). Both groups valuated the idea behind the intervention with its three potential formats: ‘It makes sense that the diary offers a review of triggers for bad days’ (healthy adult, 22, male). Patients found, ‘it helped sort my thoughts’ (patient, 22, male) and they appreciated the different choices: ‘I found it very good that the intervention has three templates. Not everyone can just start writing. Some need more structure’ (patient, 55, female). Some users, however, expressed their doubts: ‘I don't know what it takes to know that I’m annoyed’ (healthy adult, 40, female). As well, the diary intervention evoked concerns regarding data security: ‘I would not like to disclose diary entries online’ (patient, 25, female). Suggestions regarding the *usability* of the diary referred to the length of the introduction text, which was felt to be too long and features regarding the practicality of the templates on a mobile device. That a diary intervention can benefit from *personalization* features was expressed several times, for example, by choosing own colors or: ‘Maybe it would be nice if you could make the diary a little more colorful with a few emojis / GIFs / pictures. To make it more personal.’ (healthy adult, 23, female).

##### Behavioural activation

Eighty-one comments referred to the behavioural activation intervention, which was expressed 43 times by patients and 38 times by healthy adults. As this intervention was connected to the soul tank, some feedback may refer to both. The *therapeutic content* was assessed in an ambivalent way. On the one hand, the goals of the intervention were recognized and valued: ‘Taken seriously, yes! The intervention, understood as help and offer, communicates very respectfully’ (healthy adult, 22, male). On the other hand, depressed patients mentioned the dilemma of not being able to fulfil the activation encouragement: ‘During depression, the activity plan and coping strategies would not encourage me to do anything’ (patient, 55, female). Another patient wrote: ‘Just because I write something down and plan, this doesn't mean that I have managed to do it’ (patient, 36, female). This observation led the participants to suggest usability improvements: ‘How is it ensured that the plan is adhered to and is there a reminder function for the appointment?’ (patient, 36, female). Many users asked for a connection to the calendar functions of their mobile devices. Comments that were characterized as *personalization* asked for features that enhance the motivation, such as individual reminders. One of the healthy adults commented: ‘Should take me more along, … speak to me with a voice instead of having so much to read’ (healthy adult, 40, female).

##### Soul tank

The 30 comments regarding the feature *soul tank* were given only by patients, who gave a critical evaluation of the *therapeutic content* of this intervention: ‘Because I need all of my energy during a depression to cope with my everyday life to some extent. Everything that comes in addition, like in the soul tank, I can no longer manage because it is too much for me’ (patient, 55, female). Several users pointed out that they wanted to be more flexible in the number of items to put into the soul tank and in the kind of activities in the dropdown list with suggestions. ‘As if 3 small events can fill a soul tank?’ (patient, 21, female). The usability of this intervention was rated low, as it was felt to be too short, that there were not enough explanations given for guidance and the design ‘not appealing’. A possible feature to personalize the soul tank could be ‘a balance scale between negative and positive activities to represent the balance’ (patient, 23, female).

##### Album of strengths and weaknesses

All 19 comments regarding the album of strengths and weaknesses were made by patients only, who were skeptical about its *therapeutic content*. There was a strong demand for guidance, as one patient pointed out: ‘I’ve done that before in therapy. Without therapy, I would have thought of countless weaknesses and, with luck, 1–2 strengths’ (patient, 26, female). Similarly, others hinted at the ‘risk of getting lost in weaknesses’ (patient, 51, male), and: ‘I write, why I am actually okay without believing in it. I write down my weaknesses and I feel like shit. Then I look at what has been written and I have no idea how that should help me now’ (patient, 26, female). The comments that were categorized as *usability* as well revealed a wish for orientation by applying buttons to move forward or the precision of the explanations. No comments referred to *personalization*.

##### Switch off! (Radio of negative thoughts)

The intervention *Switch off!* was commented 13 times, 10 times by patients and 3 times by healthy adults. The *therapeutic content* of this intervention was felt to be too abstract and to lack a clear connection to the daily life of the participants, as one patient wrote: ‘I don't want to read through strange problems’ (healthy adult, 55, female). The concept of the intervention was criticized: ‘Often no answers apply. No “solutions” are presented, only the “problem”. That doesn't help me, because I already know that something is bad’ (healthy adult, 26, female). Comments referring to the *usability* of this intervention revealed a need for more guidance, as the users did not know when exactly this intervention was over. Potential *personalization* was suggested by comments that were coded at the same time as content: ‘Solutions how you can improve, how you can work on yourself … fewer cases, more specific and more detailed - you can then choose what applies to you’ (healthy adult, 26, female).

## Discussion

### User experience and acceptance of SELFPASS

This study asked for the user experience and the acceptance of patients and healthy adults towards SELFPASS, a symptom-specific self-management app for depression with and without anxiety symptoms. User experience was collected as perception of quality and of practicality, which both showed positive results for the whole sample. However, while the quality of the total programme (PQTP) was rated higher by patients than by healthy adults, there was no difference in the perception of P. Nevertheless, patients rated the acceptance higher than healthy adults. Acceptance in turn was associated with increasing depression scores, the PQTP and its P. Finally, the higher the acceptance, the lower the participants rated their computer skills.

Especially the latter result reflects the role of users' expectations regarding an e-mental health app, as users with good computer skills might expect potential features with a higher level of technological sophistication than available in the app. Taking the fact into consideration that depressed patients in former studies seem to be less able to concentrate on complex website presentations^31^ and to show an impaired perception of content,^30^ our results highlight the suitability of the app for the specific target group. In our sample, patients showed less computer skills than the healthy controls which explain the negative association of depression scores and the respective level of acceptance towards the app. This might as well be associated with the aspect that the healthy controls in our populations had a higher educational background. Future study concepts have to provide more elaborate study designs evaluating the exact role of the level of pre-existing digital skills. This might even win in importance as future e-mental health interventions have to meet the needs of elder patients.^58^ Asked for expectations towards an ideal e-mental health app, a former survey of the authors revealed the relevance of properties adaptable to individual needs of the users, which were more important for patients than for medical experts.^5^ Customization features and more variety were a substantial part of the propositions derived from qualitative feedback in this study.

On the level of single interventions, no significant differences between patients and healthy adults were observed. Both groups appreciated the possibility of journal keeping, which is supported by the literature about similar approaches of online writing.^59^ Regarding other main interventions, such as relaxation and concentration, the need for choice and adaptation of single functions was obvious.

In line with this, the uptake and successful use of the app for daily usage in the future depends on balancing customization features, which depend on personal data tracking,^41^ and the transparent enforcement of strict information security measures protecting such personal data. This requirement, which was expressed several times by the participants in the present investigation, is highlighted by available mental health apps as well. Accordingly, a recent study in 2019 with 61 prominent examples showed that only half of the apps informed their users about their privacy policy.^60^

Besides from this, we see another challenge for developers and clinicians: In our study, participants wanted for guidance, especially depressed patients, who felt being left alone with the software. This was accompanied by the necessity for transparent assessment results and for understanding the therapeutic rationale for intervention recommendations. Today, patients want to be involved in their treatment process, to acquire health literacy, and be included in shared decision-making.^61^ Again, the demand for more choice and customization will obviously be accompanied by a steady increase of other users alongside the technological advancement of mobile technology. Moreover, many patients reported not to believe in sustainable positive effects of the interventions, which might be a result of their depressive disorder due to the attentional bias to negative thinking.^62^ Future research might focus on the interaction of technology use and potential interventions to tackle this problem.

As elaborated in the beginning, available e-mental health apps do not fully draw on current technological capabilities.^17^ Although mood tracking and symptom monitoring are implemented in the majority of depression apps,^18,22,40^ patients' demands for customization in depression apps are still unmet.^63^ For example, EMA, such as applying step counts, assessing movement profiles, and simultaneously analysing the patients' mood, bears a great potential for treatment, but it is only implemented in scientific studies up to now.^35^

### Recommendations for developers and clinicians

In line with our results and the literature reviewed, we summarized some recommendations for developers and clinicians who are working on future e-mental health applications. By this, we hope to contribute to close the gap between high-level scientific e-mental health apps and the low real-world uptake and user engagement up to now.^29,64^

1. *Personalization*: Allow users to customize and build their own interventions (e.g. upload of pictures, adaptable levels, text messages, rating).
2. *Transparency*: Inform the users about their results, the reasons for the treatment recommendations and the therapeutic background of interventions.
3. *Data security*: Implement a state-of-the-art data security concept and keep users informed about it.
4. *Design*: Take patients seriously. Do not offer a childlike language in intervention introductions or emoticons in mood tracking questions.
5. *Gamification*: Keep users motivated by delivering gamification elements, for example, by collecting points, while respecting the seriousness of the topic (see design).
6. *Structure*: Find the right balance between guidance and personalization. Patients do not want to feel being left alone with an app. Instead, they want to be led through a programme and be informed – transparently – about decisions made by the programme. For example, reminders for the completion of daily activities are a helpful opportunity in providing guidance.
7. *Crisis management*: Be aware of the limits of technology and provide an efficient concept of crisis management with a realistic contact scenario in case of suicidality.

### Study limitations

Some limitations occurred during the study that should be considered. First, the recruitment of the participants was done in two very different time spans. The second recruitment period suffered from restrictions due to the COVID-19 pandemic. Therefore, we could only contact the participants via telephone, which might have influenced the adherence to the study goals. Second, some healthy adults were less reliable in filling out the whole questionnaire. However, this led us to the decision to pay attention to the qualitative assessment of those, who obviously wanted to contribute to the further development of the app SELFPASS. Moreover, we were not able to match patients and healthy adults regarding all characteristics which led to differences in the respective educational background and their computer skills. We did not ask for medication or treatment history, as patients in psychosomatic services suffer from complex comorbidities. Finally, we avoided doing Bonferroni adjustments, as the multiple comparisons in the descriptive data only served as exploratory testing for a deeper understanding of the sample characteristics.

## Conclusions

Patients with depression accepted the app SELFPASS more than healthy adults and gave higher quality ratings. User experience of all users reveals a strong need for features of guidance, choice and customization. Clinicians and developers of future apps should pay attention to the special needs of patients with depression to increase their adherence and treatment success.

## Supplemental Material

sj-docx-1-dhj-10.1177_20552076221091353 - Supplemental material for User experience and acceptance of patients and healthy adults testing a personalized self-management app for depression: A non-randomized mixed-methods feasibility study Click here for additional data file. Supplemental material, sj-docx-1-dhj-10.1177_20552076221091353 for User experience and acceptance of patients and healthy adults testing a personalized self-management app for depression: A non-randomized mixed-methods feasibility study by Gwendolyn Mayer, Svenja Hummel, Neele Oetjen, Nadine Gronewold, Stefan Bubolz, Kim Blankenhagel, Mathias Slawik, Rüdiger Zarnekow, Thomas Hilbel and Jobst-Hendrik Schultz in Digital Health
