# Supplementary material for: User experience and acceptance of patients and healthy adults testing a personalized self-management app for depression: A non-randomized mixed-methods feasibility study
Source: Digit Health. 2022 Apr 7;8:20552076221091353. doi: 10.1177/20552076221091353 (PMC9003643; doi:10.1177/20552076221091353)
Supplement: sj-docx-1-dhj-10.1177_20552076221091353 - Supplemental material for User experience and acceptance of patients and healthy adults testing a personalized self-management app for depression: A non-randomized mixed-methods feasibility study [file sj-docx-1-dhj-10.1177_20552076221091353.docx]

Appendix: Assignment of interventions based on depressive or anxious symptoms

| **No.** | **Intervention** | **Relevant Symptom*** |
| --- | --- | --- |
| 1 | journal keeping | all |
| 2 | behavioral activation | CS 1-3 |
| 3 | thought stop | AS2-4, AS6 |
| 4 | cognitive restructuring | CS1-2, AS2-4 |
| 5 | album of strengths and weaknesses | CS1, AS2, CA |
| 6 | concentration exercise | AS1 |
| 7 | psychoeducation | all |
| 8 | soul tank | CS2-3, AS4 |
| 9 | relaxation exercise: “My safe place” | CS1, CA |
| 10 | relaxation exercise: "Laying down a burden" | CS3, AS2-3 |
| 11 | relaxation exercise: "Meeting my inner power" | CS3, AS2, AS4 |
| 12 | relaxation exercise: "Making peace with myself" | CS1, AS3 |
| 13 | calming exercise (“Come to the ground”) | CA |
| 14 | coping intervention (letter to oneself) | AS2-4, CA |
| 15 | emergency contact | AS5 |

Symptoms (ICD-10):

CS: core symptom

1. depressed mood
2. loss of interest
3. loss of energy

AS: additional symptom

1. lack of concentration
2. feelings of worthlessness
3. guilt
4. pessimistic future expectations
5. suicidal ideation
6. sleep disturbances
7. loss of appetite

CA: comorbid anxiety
